# Supplementary material for: Causal Relationship between Aging and Anorexia Nervosa: A White-Matter-Microstructure-Mediated Mendelian Randomization Analysis
Source: Biomedicines. 2024 Aug 16;12(8):1874. doi: 10.3390/biomedicines12081874 (PMC11351342; doi:10.3390/biomedicines12081874)
Supplement: Supplementary file 1 [file biomedicines-12-01874-s001.zip › Supplementary Figure/Figure S7/Single SNP plot of MR analysis for ubm-b-1954 to AN.pdf]

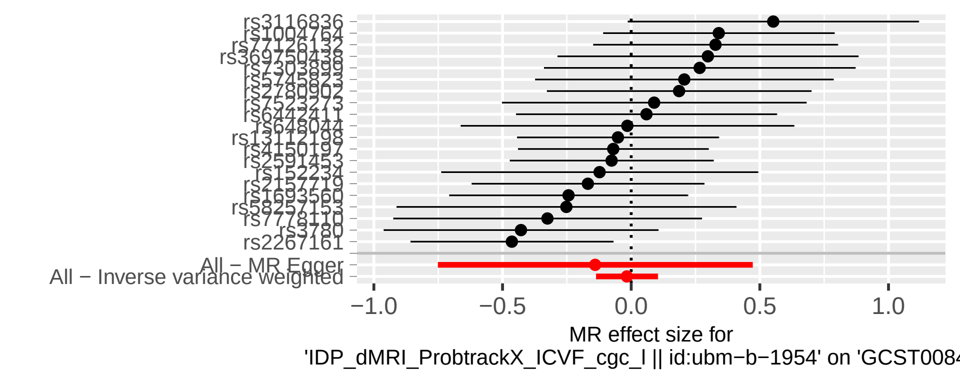

rs3116836
rs1004764
rs77126132
rs369750436
rs7303899
rs5745823
rs2780902
rs7523273
rs6442411
rs648044
rs13112198
rs4150197
rs2591453
rs152234
rs2157719
rs1693560
rs58257153
rs7778110
rs3780
rs2267161
All − MR Egger
All − Inverse variance weighted
−1.0
−0.5
0.0
0.5
1.0
MR effect size for
'IDP_dMRI_ProbtrackX_ICVF_cgc_l || id:ubm−b−1954' on 'GCST008
